# Plasma Cytokine Levels Fall in Preterm Newborn Infants on Nasal CPAP with Early Respiratory Distress

**DOI:** 10.1371/journal.pone.0120486

**Published:** 2015-03-23

**Authors:** Clarissa Gutierrez Carvalho, Rita de Cassia Silveira, Eurico Camargo Neto, Renato Soibelmann Procianoy

**Affiliations:** Department of Pediatrics, Universidade Federal do Rio Grande do Sul, and Newborn Section, Hospital de Clínicas de Porto Alegre, Porto Alegre, Rio Grande do Sul, Brazil; The Ohio State Unversity, UNITED STATES

## Abstract

**Introduction:**

Early nCPAP seems to prevent ventilator-induced lung injury in humans, although the pathophysiological mechanisms underlying this beneficial effect have not been clarified yet.

**Objective:**

To evaluate plasma levels IL-1β, IL-6, IL-8, IL-10, and TNF-α immediately before the start of nCPAP and 2 hours later in preterm infants.

**Methods:**

Prospective cohort including preterm infants with 28 to 35 weeks gestational age with moderate respiratory distress requiring nCPAP. Extreme preemies, newborns with malformations, congenital infections, sepsis, surfactant treatment, and receiving ventilatory support in the delivery room were excluded. Blood samples were collected right before and 2 hours after the start of nCPAP.

**Results:**

23 preterm infants (birth weight 1851±403 grams; GA 32.3±1.7 weeks) were treated with nCPAP. IL-1β, IL-10, TNF-α levels were similar, IL-8 levels were reduced in 18/23 preterm infants and a significant decrease in IL-6 levels was observed after 2 hours of nCPAP. All newborns whose mothers received antenatal steroids had lower cytokine levels at the onset of nCPAP than those whose mothers didn’t receive it; this effect was not sustained after 2 hours of nCPAP.

**Conclusion:**

Early use nCPAP is not associated with rising of plasma pro-inflammatory cytokines and it seems to be a less harmful respiratory strategy for preterm with moderate respiratory distress.

## Introduction

The early use of mechanical ventilation (MV) has been shown to induce pro-inflammatory cytokine expression [1,2]. Noninvasive ventilation and early initiation of continuous positive airway pressure (CPAP) in the delivery room seem to be promising strategies to prevent MV-induced injury in extremely preterm infants [3–5].

Individual trials of early CPAP therapy versus invasive ventilation in the delivery room [4–5] have not resulted in a reduced rate of bronchopulmonary dysplasia (BPD), but pooling data on combined mortality and BPD has suggested a benefit [6]. CPAP seems to facilitate spontaneous breathing, maintain alveolar recruitment, and reduce the need for MV in preterm infants [4]—pathophysiological mechanisms underlying the beneficial effects of CPAP have not yet been clarified and may be due mainly from the avoidance of invasive ventilation or inadvertent exposure to high tidal volumes and hyperventilation [7].

The mechanisms behind the protective effect of nasal CPAP (nCPAP) in ventilator-induced lung injury (VILI) have only been studied in animals [8]. One study designed to analyze cytokine levels in the first week of life in non-infected infants using nCPAP showed some small median decrease between blood samples collected on birth and until 4 hours of life [9]. Lista el al. [10] compared cytokine levels between preterm newborns at the first and seventh day of nCPAP or nasal intermittent positive pressure ventilation (NIPPV), suggesting that both ventilatory modes are protective. It seems that the use of nCPAP, even for a short period of time, might prevent harmful pro-inflammatory stimuli to the lung.

Thus, the aim of this study was to determine plasma levels of interleukin (IL)-1β, IL-6, IL-8, IL-10, and tumor necrosis factor (TNF)-α in preterm newborns in their first hours of life, right before start of nCPAP and 2 hours later.

## Methods

A prospective cohort study included preterm infants admitted between September 2011 and May 2013 to the neonatal intensive care unit (NICU) of Hospital de Clínicas de Porto Alegre, a tertiary referral medical center located in Southern Brazil. Gestational age (GA) ranged from 28 to 35 weeks and all participants used nCPAP as the initial support in the first 24 hours of life. Exclusion criteria were extreme preemies, congenital malformations or chromosomal syndromes, STORCH infections, proven sepsis, meningitis, need for ventilatory support with any type of positive airway pressure therapy in the delivery room, and use of nitric oxide and surfactant prior to enrollment in the study.

Study was approved by Research Ethics Committee of Hospital de Clinicas de Porto Alegre (project number 11–0325). Written informed consent was obtained from the parents or guardians prior to enrollment of participants.

The following data were collected: gestational age (based on the date of the last period and confirmed by ultrasound in the first trimester and/or neonatal clinical examination), birth weight, gender, Score for Neonatal Acute Physiology Perinatal Extension II (SNAPPE-II), delivery room resuscitation, type of delivery, and presence of preeclampsia, amniorrhexis, and/or chorioamnionitis. Preterm infants whose mothers had received two doses of betamethasone prior to delivery were considered to have received antenatal steroids. Moderate respiratory distress was defined by the presence of grunting, tachypnea, respiratory effort and oxygen need.

Newborns were followed from birth to the start of nCPAP, blood samples were collected for arterial blood gas analysis according to the routine of the NICU, and an additional 500μL aliquot was obtained in Ethylenediamine tetraacetic acid (EDTA) tubes for later cytokine analysis. No additional venous or arterial blood punctures were performed strictly for research purposes. After two hours on nCPAP, another sample was collected for arterial blood gases and cytokine levels. The blood samples were immediately centrifuged for 10 minutes at 3,000 rpm to yield 300 μL of plasma, which was frozen at—80°C for later laboratory analysis of cytokines. Number and time of collection identified all samples.

Cytokines were measured using a commercially available kit (MILLIPLEX Human Cytokine/Chemokine MPXHCYTO-60K, Millipore Corporation, Billerica, MA USA). The readings were performed with Luminex 100 in duplicate (Austin, Texas, USA) with appropriate software. Samples and standard curve were processed

The alveolar-arterial oxygen gradient—P (Aa) O_2_—and the arterio-alveolar oxygen ratio (a/ApO_2_) were calculated from arterial blood gas analyses before and 2 hours after the start of nCPAP.

### Statistical analysis

Sample size was calculated based on a previous study with animals [8], which compared three groups regarding to cytokine increase after a two-hours interval blood collection—MV, CPAP and none. To test the hypothesis that nCPAP does cause an increase of 37% in cytokine levels during its use, which would be smaller than MV increase, 19 newborns would be required (α = 0.05 and 80% power). The results were expressed as mean ± standard deviation (SD) or median and range (p25-p75). The Wilcoxon signed-rank test was used to compare cytokine levels immediately before the onset and 2 hours after nCPAP. Mann-Whitney’s test was used for the other comparisons. A delta value between pre and post nCPAP was created for each interleukin and transformed to log10 for additional testing, allowing logistic regression performing and parametric tests. Analyses were performed with Statistical Package for Social Sciences (SPSS), version 18.0, and the level of statistical significance was p <0.05.

## Results

Twenty-three newborns met the inclusion criteria. Of these, 7 (30%) patients were small for gestational age, 13 (56%) males, 18 (78%) delivered by C-section and 6 (23%) delivered by preeclamptic mothers. A full antenatal steroid course was given to 15 mothers (65%). Mean birth weight was 1851±403 grams, and mean gestational age was 32.3±1.7 weeks. Median SNAPPEII score was 7 (0–9). The median time for collection of samples for cytokine measurement and nCPAP onset was 2 (1.5–2.5) hours of life.

The median alveolar-arterial oxygen gradient was lower after 2 hours on nCPAP: prior = 62.4 mmHg (33.2–105.8) and after = 33.6 mmHg (13–70) (p = 0.057).

**Fig 1:**
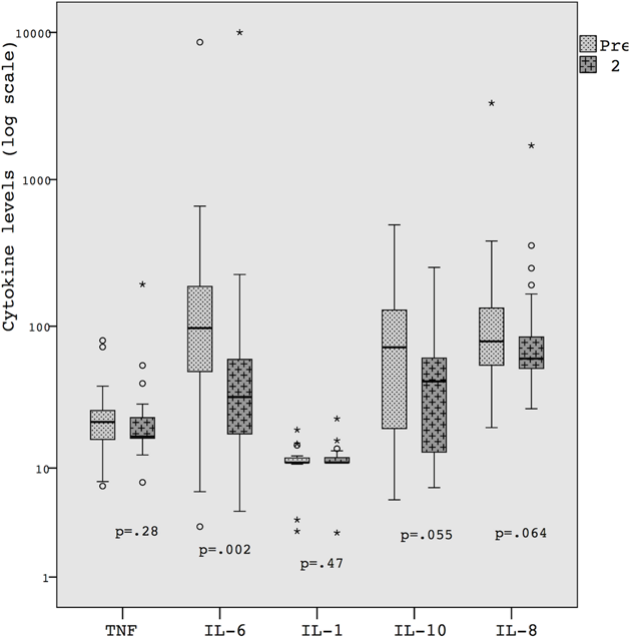
Cytokine levels immediately after the onset of nCPAP and after 2 hours—Wilcoxon’s test.

IL-6 median plasma levels were significantly reduced after 2 hours of nCPAP (Table 1 and Fig. 1). IL-8 levels were reduced after 2 hours of nCPAP in 18 of 23 preterm infants (78%), as well as TNF- α in 13 (56%), and IL-10 in 16 (69%) and only four patients showed a reduction in IL-1β.

**Table 1:** Cytokine median levels immediately before onset of nCPAP and after 2 hours.

Cytokine (pg/dL)	Pre-nCPAP	After 2 hours n-CPAP	p
**N**	**23**	**23**	
**IL-6**	97.1 (43.1–188)	32.4 (17.6–62)	**0.002**
**IL-8**	78.8 (52.8–133.8)	59.9 (51–87.5)	0.064
**IL-10**	71.6 (14–145)	41.7 (13–63.2)	0.055
**IL-1β**	11 (11–11.9)	11 (11–12)	0.47
**TNF-α**	21.6 (15.7–26.4)	17 (16.5–23.7)	0.28

Median (p25-p75).

Wilcoxon signed-rank test.

**Fig 2:**
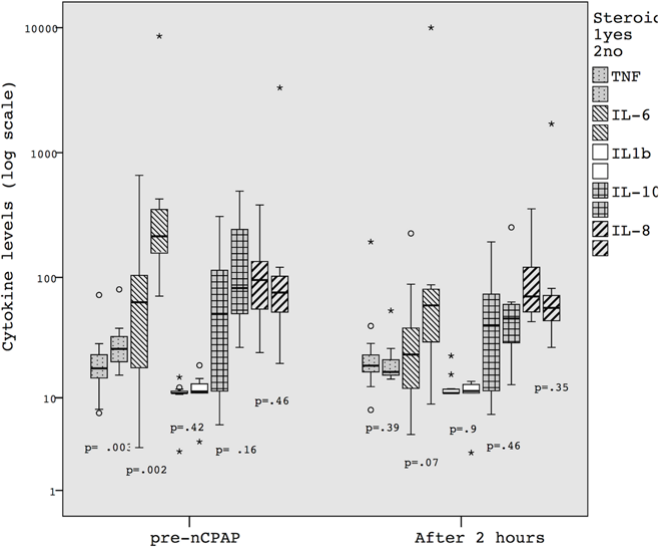
Comparing cytokine levels regarding to antenatal steroid use immediately before the onset of nCPAP and after 2 hours, Mann-Whitney test.

The median plasma levels of pre-nCPAP IL-6 and TNF-α were lower in the 15 infants whose mothers received antenatal steroids. However, this effect was not sustained after 2 hours of nCPAP (Table 2 and Fig. 2). After logistic regression, there was no relationship regarding post-natal age in hours (IL-6 OR 0,6; IC 95% 0,15–2,3, TNF- α OR 0,3; IC 95% 0,07–1,8), delivery mode (IL-6 OR 2,1; IC 95% 0,2–21, TNF- α OR 0,6; IC 95% 0,05–6,8) and cytokines decrease (IL-6 OR 6,2; IC 95% 0,5–66, TNF- α OR 73; IC 95% 0,5–10000).

**Table 2:** Impact of antenatal steroid use on cytokine levels immediately before the onset of nCPAP and after 2 hours.

Cytokine (pg/dL)	IL-6			IL-8			IL-10			IL-1β			TNF-α		
Antenatal steroid	Pre-CPAP	After	p *	Pre-CPAP	After	p *	Pre-CPAP	After	p *	Pre-CPAP	After 2h	p *	Pre-CPAP	After 2h	p *
		2h			2h			2h							
**Yes**	63	23	**0.009**	95	70	0.17	50.5	40	0.53	11	11	0.12	18	19	0.53
**(n = 15)**	(15–111)	(8–45)		(55–135)	(51–166)		(10.5–114)	(11–94)		(11–12)	(11–12)		(15–24)	(16–24)	
**No**	216	59	0.161	75	57	0.12	82	46.5	**0.02**	11	11.5	0.46	26	17	**0.01**
**(n = 8)**	(149–392)	(26–84)		(52–112)	(42–77)		(49–327)	(26–62)		(11–14)	(11–13)		(19–36)	(15–24)	
**p”**	**0.002**	0.076		0.46	0.35		0.16	0.46		0.42	0.9		**0.034**	0.39	

* Median (p25-p75), Wilcoxon signed-rank test.

“Median (p25-p75), Mann-Whitney test.

## Discussion

A protective and/or inhibiting effect of early nCPAP on the pro-inflammatory cascade in preterm newborns is suggested in this study. IL-6 plasma levels were significantly lower after two hours of nCPAP; and both IL-8 and TNF- α levels were reduced in more than 50% of preterm infants after two hours of nCPAP—all of those are proinflammatory cytokines involved in VILI.

Animal studies have shown that positive end-expiratory pressure (PEEP) ventilation reduced edema formation and cell damage [7], as well as inflammatory cell recruitment, during prolonged ventilation [11]. However, even the available experimental data are controversial: preterm lambs receiving tracheal CPAP presented only slightly lower cytokine levels after 2 hours as compared to those receiving MV [8]. Similar increased levels of IL-1β were recorded with both ventilation modes, whereas IL-6 and IL-8 were elevated only in the MV group [12]. Conversely, Polglase et al. [13], using a lipopolysaccharide infection model, did not observe decreased inflammatory response with the use of CPAP, suggesting a limited effect of this ventilation mode in the presence of both immature lungs and infectious injury. Timing of sample collection is an important factor in the evaluation of inflammatory response; cytokines half-life is very short, and their circulating levels increase rapidly after stimulation [14]. The strength of our study is that the collection of samples prior to and 2 hours after the stimulus provided a safe interval to prevent the onset of other potential inflammatory events, which are characteristic of preterm newborns and that could interfere with or alter the exclusive role of nCPAP on inflammatory cascade.

Procianoy et al. [14] observed that IL-6 levels, in newborns which blood was collected as early as 17 hours of life, were higher than in those who collected at 36 hours, suggesting a decrease in cytokine levels late in the first 24 hours of life. Other studies demonstrated that IL-6 levels increased after birth until 24 hours of life in healthy neonates [15,16], decreasing over the following second day. A randomized clinical trial comparing nCPAP with no nCPAP or nCPAP with mechanical ventilation would be the ideal study design but each ventilatory therapy has its own precise indication. Despite the absence of a non-nCPAP control group—justified by ethical issues involving blood collection in patients that do not need an exam—we believe our results do not represent the natural course of cytokines release.

Because IL-6 is the main cytokine involved in the development of the fetal inflammatory response syndrome [17], it has been described as an early marker of BPD [18] and neonatal sepsis [19, 20]. Therefore, we excluded all cases of neonatal sepsis and STORCH infections, as well as neonates requiring any type of ventilatory support in the delivery room, who might have elevated baseline IL-6 levels—a situation that might impact the evaluation of the behavior of IL-6.

Interestingly, the levels of inflammatory cytokine IL-10 were reduced in 69% of the newborns 2 hours after nCPAP. Anti-inflammatory expression of IL-10 occurs later [21], after the release of IL-8, especially in preterm infants with gestational age below 30 weeks [22]. The production of down regulatory interleukins, such as IL-10, may be insufficient in preterm newborns, which leaves them more predisposed to an exacerbated inflammatory response [23]. The very low levels of IL-1β may also be explained by kinetics, since the rate of IL-1β increase is slower in the first 24 hours of life, the period in which all our samples were obtained [24].

In a previous study, preterm newborns with less than 35 weeks of gestational age had similar levels of IL-6, IL-8, and TNF-α at the first and seventh day of nCPAP or NIPPV, which suggests that both ventilatory modes are protective—evaluating a sustained inflammatory response [10]. Our data shows that after a short period of only a couple of hours, the inflammatory response associated with protective ventilation is significantly different.

The inhibitory action of antenatal steroid administration on inflammatory response and ventilation-induced injury is well known [25]. Using antenatal steroid in women during preterm labor has been associated with a significant reduction in the baseline production of IL-6 in the umbilical cord [26]. Our preterm newborns receiving nCPAP who had been exposed to intrauterine steroids had significantly lower pre-nCPAP IL-6 and TNF-α plasma levels as compared to non-exposed newborns. However, the levels of these markers were similar in all nCPAP newborns two hours later, showing a transient anti-inflammatory action of antenatal steroid administration.

The improvement in gas exchange after nCPAP demonstrated a proper use of this support to moderate respiratory stressed newborns. We did not include extremely premature newborns, with gestational age below 28 weeks, because the success rate in applying nCPAP in that group was low in our service, during the study period. Extremely preterm newborns are more often treated with MV and affected by systemic inflammation than other preterm populations; however, their exclusion from the present study is also justified by the fact that these baseline inflammations would possibly impact interleukin levels [27]. Another study limitation was our small sample size, with enough power to detect some change in cytokine levels but unable to test other clinical relevant outcomes. A larger sample size would be necessary to draw more conclusions.

## Conclusion

We suggest that early use nCPAP is not associated with release of plasma pro-inflammatory cytokines, especially considering the reduction in IL-6 plasma levels. Thus, our results propose that nCPAP is a less harmful initial ventilatory strategy for preterm infants with moderate respiratory distress.
